# A cognitive perspective on health systems integration: results of a Canadian Delphi study

**DOI:** 10.1186/1472-6963-14-222

**Published:** 2014-05-19

**Authors:** Jenna M Evans, G Ross Baker, Whitney Berta, Jan Barnsley

**Affiliations:** 1 Institute of Health Policy, Management & Evaluation, Faculty of Medicine, University of Toronto, Health Sciences Building, 155 College Street, Suite 425, Toronto, Ontario M5T3M6, Canada

**Keywords:** Health systems integration, Integrated care, Shared mental models, Group cognition, Organizational culture, Change management

## Abstract

**Background:**

Ongoing challenges to healthcare integration point toward the need to move beyond structural and process issues. While we know what needs to be done to achieve integrated care, there is little that informs us as to *how*. We need to understand how diverse organizations and professionals develop shared knowledge and beliefs – that is, we need to generate knowledge about *normative integration*. We present a cognitive perspective on integration, based on shared mental model theory, that may enhance our understanding and ability to measure and influence normative integration. The aim of this paper is to validate and improve the Mental Models of Integrated Care (MMIC) Framework, which outlines important knowledge and beliefs whose convergence or divergence across stakeholder groups may influence inter-professional and inter-organizational relations.

**Methods:**

We used a two-stage web-based modified Delphi process to test the MMIC Framework against expert opinion using a random sample of participants from Canada’s National Symposium on Integrated Care. Respondents were asked to rate the framework’s clarity, comprehensiveness, usefulness, and importance using seven-point ordinal scales. Spaces for open comments were provided. Descriptive statistics were used to describe the structured responses, while open comments were coded and categorized using thematic analysis. The Kruskall-Wallis test was used to examine cross-group agreement by level of integration experience, current workplace, and current role.

**Results:**

In the first round, 90 individuals responded (52% response rate), representing a wide range of professional roles and organization types from across the continuum of care. In the second round, 68 individuals responded (75.6% response rate). The quantitative and qualitative feedback from experts was used to revise the framework. The re-named “Integration Mindsets Framework” consists of a Strategy Mental Model and a Relationships Mental Model, comprising a total of nineteen content areas.

**Conclusions:**

The Integration Mindsets Framework draws the attention of researchers and practitioners to how various stakeholders think about and conceptualize integration. A cognitive approach to understanding and measuring normative integration complements dominant cultural approaches and allows for more fine-grained analyses. The framework can be used by managers and leaders to facilitate the interpretation, planning, implementation, management and evaluation of integration initiatives.

## Background

Innovative healthcare delivery models often incorporate the concepts of integration and integrated care. By bringing together multiple professionals, services, and organizations, integration efforts aim to replace fragmented care with care that is coordinated and patient-centered [1]. Despite the promise of improved efficiency, quality of care, and patient satisfaction, healthcare systems capable of delivering integrated care have not developed widely [2-4].

Over the past two decades, most scholars have focused on the structural and process challenges involved in integrating care. The resulting body of knowledge on integration barriers and enablers has informed many positive system changes. Progress has also been made in understanding, effecting, and evaluating various types of integration, including organizational, functional, service, and clinical integration [5]. However, reports continue to highlight the seemingly intractable problems inherent in fostering collaboration and cooperation across professional and organizational boundaries [6-9]. These problems point toward the need to move beyond the integration of organizational structures and processes to understand the social cognitions that are implicated in integration efforts. *Normative integration*, defined as “an ethos of shared values and commitment to coordinating work [which] enables trust and collaboration in delivering healthcare”, may be the key to addressing ongoing challenges to integrated care delivery [5]; but we still know little about how to achieve and measure normative integration.

One way to explore normative integration is by examining organizational and professional cultures, defined as the beliefs and behaviour patterns dominant among a group of people [10]. For example, at the outset of the implementation of an integration initiative one may gauge the extent to which there are discrepancies among cultures that will need to be addressed. In turn, post-implementation cultural harmonization may be used as evidence of normative integration. Although culture is rarely directly measured in studies of integration, cultural differences are often offered as explanations for failed or suboptimal integration initiatives [8,11,12]. Despite this popular attribution, there is little evidence on the effectiveness of strategies to change culture, and debates persist regarding the feasibility and desirability of merging various cultures in healthcare [13,14].

An alternative perspective is to view integration through the lens of individual and shared mental models. Mental models are psychological representations, consisting of knowledge and beliefs, which enable interpretation and action in a specific domain [15]. Mental models are developed over time through experience, direct communication and interaction with others, and vicarious learning [16]. When multiple individuals develop a common psychological structure for understanding their environment, this is referred to as a Shared Mental Model (SMM) [15,17]. Mental models inform perception and behavior, and shape culture in a co-evolutionary process [10,18,19]. Like cultural congruence, mental model similarity may also be used as an indicator of readiness to integrate, or post-implementation similarity as a sign of successful integration. While both constructs, culture and SMMs, help to describe and explain normative integration, a mental models perspective may allow for more fine-grained analyses of inter-organizational and inter-professional relations [20]. Mental models may also be more amenable to change than cultures and SMMs may be a means for bridging diverse cultures [19,21].

Drawing from an interdisciplinary literature review and SMM theory, Evans and Baker [20] developed a framework of mental model content, antecedents, and outcomes *specific* to integration initiatives in the healthcare sector. Their framework outlines important knowledge and beliefs whose convergence or divergence across stakeholder groups may influence inter-professional and inter-organizational relations. This study builds on that work, and tests and improves the Mental Models of Integrated Care (MMIC) Framework through an iterative process using expert opinion. An expert-validated framework will support the application of a cognitive perspective in future research and practice on healthcare integration.

### Theoretical framework

In this study, we draw from Shared Mental Models (SMMs) theory, which posits that team effectiveness is maximized when members of a team have a shared understanding of their tasks and roles [15]. SMMs fall into three broad categories: *task-related* (goals and performance requirements), *team-related* (interpersonal interaction requirements and skills of team members), and *beliefs* (preferences or expectations) [15]. SMMs in these content areas improve team and organizational performance by facilitating coordinated action and adaptation under changing conditions [15,22-24]. SMMs allow individuals to develop common views of what is happening, what is likely to happen next, and why it is happening, and thus guides their behaviours in ways that are consistent and coordinated with each other in the completion of interdependent tasks [15]. Although diversity in perspectives has been linked to high decision quality and improved team performance, this is likely due to the positive effects of dissent at the strategy formulation or decision-making stage; at the point of action or implementation, however, SMMs positively impact performance [25]. That being said, *identical* knowledge and beliefs are not necessary; convergence around a broad frame of mental models provides the common meaning needed for action, regardless of differing views on specific issues [26]. Thus, we define “shared” as *similar* and *overlapping*, not identical, mental models among a group of individuals.

Research in strategic management, stakeholder management, change management, systems change, and team, organizational and network performance also emphasize the importance of developing common aims and shared understandings e.g., [27-29]. Although SMMs are group-level phenomena, the extent to which individual perspectives and behaviours are consistent (or not) can impact performance *beyond* the group level [28,30,31]. For example, in a study examining healthcare personnel’s mental models of the organizational implementation of clinical practice guidelines, personnel in high-performing facilities exhibited SMMs, while those in lower performing facilities did not [30]. SMMs help to shape broader constructs such as inter-organizational macro-cultures, industry mindsets, and institutional logics, which also suggests that SMMs can manifest at the systems level [32-34]. Hence, SMMs may facilitate the implementation of complex system-level change involving the inputs of a multitude of diverse actors. Systems integration, which aims to link various sectors, organizations and professionals across the continuum of care, is a prime example of such a change. For a more thorough justification of the application of SMMs theory to healthcare integration and a discussion of strengths and limitations, please refer to Evans and Baker [20].

Applied to integration, the three mental model categories are integration-task, system-role, and integration-belief [20]. An integration-task mental model encompasses the purpose and approach to integration and includes the following contents: services to be integrated; external customers; goals; long-term vision; and processes. A system-role mental model refers to one’s understanding of the system and its components, and includes the following contents: knowledge, skills and abilities of participating professionals and organizations; role clarity; role interdependence; role contribution; and interaction patterns. Finally, an integration-belief mental model includes relevant preferences and expectations as well as meanings, assumptions, and interpretations of key issues. This initial framework was derived from a review of previous theory and research [20]. In this paper, we examine the extent to which the Mental Models of Integrated Care (MMIC) Framework, and its dimensions and contents, are relevant, acceptable and useful to those with expertise and experience in integration efforts, and we revise the framework based on this assessment.

## Methods

We evaluated and refined the proposed MMIC Framework using expert opinion in a two-round web-based modified Delphi process. The Delphi method is a consensus-building technique that solicits the opinions of content experts in a given field through the use of a series of questionnaires combined with the provision of feedback [35]. Using the participant list (n = 344) from the Health Council of Canada’s National Symposium on Integrated Care, we selected and invited a random sample of 172 individuals to participate in our study (50% of the population). The Symposium, held on 10 October 2012, brought together policymakers, planners, managers, care providers, educators, researchers and patient advocates from across Canada to share and promote the spread of innovative practices in integrated care.

Following the Symposium, experts were invited via email to participate in the study. They were asked to commit to completing two questionnaires (~30 minutes each) and to have access to email or the Web for receiving and returning questionnaires. Completing the questionnaires implied consent to participate. Respondents were assured confidentiality, but not anonymity. Coffee cards ($10 each) were offered as an incentive for participating in both rounds of the study. For each round, two reminders were sent to non-respondents via email. The study received ethics approval from the Office of Research Ethics at the University of Toronto (protocol #28076).

The questionnaire was developed and designed using Dillman’s [36] criteria for question and questionnaire design with Survey Monkey as the platform. Prior to administration, the questionnaire was pre-tested with eight volunteers (two care providers, one health services researcher, and five managers from a range of healthcare organizations) for clarity, and to anticipate the average completion time. All volunteers had knowledge of and/or experience in integration initiatives. As a result of the pre-test, modifications were made to the questionnaire’s length, instructions, and lay-out. The pre-test also highlighted differences in how “integration” and “integrated care” were defined and perceived. All the volunteers preferred “mental models of integration” over “mental models of integrated care” because the latter focuses exclusively on direct patient care to the perceived exclusion of other forms of integration, such as organizational and functional integration. We changed the terminology accordingly. We also made minor modifications to two of three mental model categories based on the pre-test: “integration-task mental model” and “integration-role mental model” were replaced with “strategy mental model” and “roles mental model” respectively.

The first round of the web-based questionnaire took place between 22 October and 12 November 2012 and consisted of 46 items (Additional file 1). The questionnaire provided a two-page overview of the proposed framework, followed by questions which asked respondents to rate the clarity, comprehensiveness, usefulness, and importance of the concepts in the framework, and the framework itself, using seven-point ordinal scales. Respondent demographics were also collected and spaces for comments were provided throughout the questionnaire.

The second questionnaire, sent only to those who responded to the first round, took place between 4 December and 20 December 2012 and consisted of 33 items (Additional file 2). The questionnaire did not include questions on respondent demographics and questions on importance were not repeated due to high ratings from round one. The general format and remaining questions were the same as in the first questionnaire with three additions: (a) a summary of the feedback from round one, including response rate, participant demographics, and major findings, (b) an enhanced introductory explanation of the framework, its background, and its potential applications, and (c) quantitative and qualitative feedback from the first questionnaire for each question. The feedback included an outline and explanation of the modifications made and not made to the framework based on the first round results. The second questionnaire was pre-tested for clarity and ease of completion prior to administration with a sub-set of four volunteers from the original group of eight from round one. No changes were made to the questionnaire as a result of the pre-test.

The results for both questionnaires were tabulated using quantitative and qualitative analyses. Descriptive statistics were used to describe the structured responses as a whole, while the open comments were coded and categorized using thematic analysis to identify common themes and key issues. The Kruskall-Wallis test was used to examine cross-group agreement by level of integration experience, current workplace, and current role. In the questionnaire, respondents had the option of selecting multiple roles and workplaces; less than 5% (n = 4) and 15% (n = 12) chose to do so respectively. For the purpose of analysis, however, each respondent was assigned only one role and one workplace based on the information they provided in the questionnaire (i.e., primary affiliation).

## Results

### Round I questionnaire

**Figure 1:**
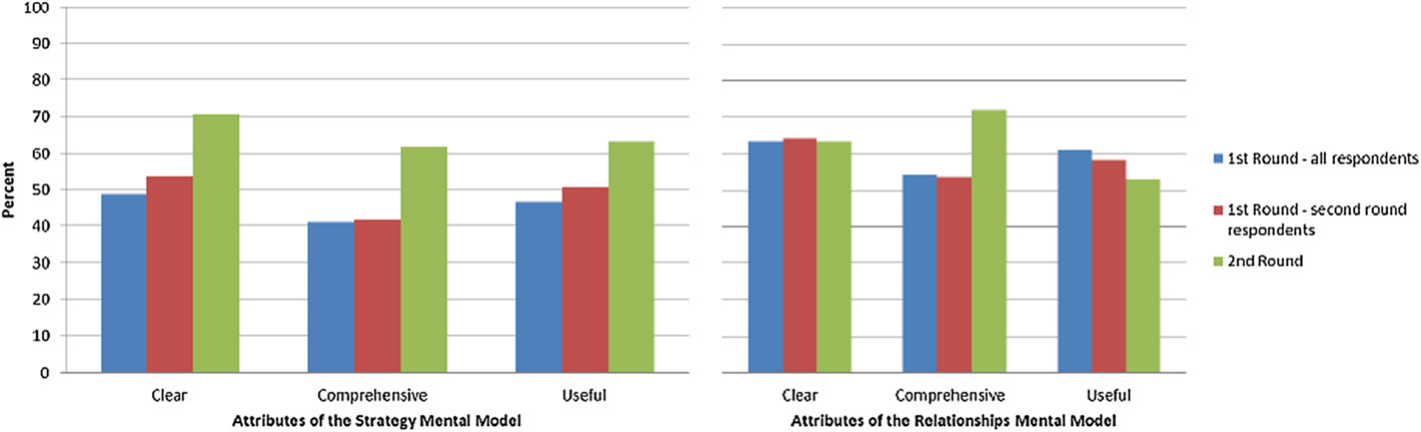
**Delphi results: Attributes of the mental models.** Are the definition, description and contents of the Strategy/Relationships Mental Model clear, comprehensive, and useful? Average percent strongly agree (rating of 6 or 7 on a 7-point scale).

**Figure 2:**
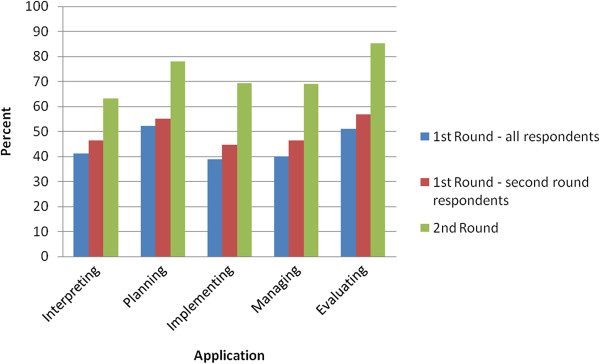
**Delphi results: Practical applications of the framework.** Is the framework, as a whole, useful for each of the following applications? Average percent strongly agree (rating of 6 or 7 on a 7-point scale).

In the first round of the Delphi process, a total of 90 individuals responded from nine provinces across Canada (52% response rate). As outlined in Table 1, respondents represented a wide range of roles and organizations in the healthcare system, and 96.7% had direct experience in integration activities. Over 250 comments were made in total. The responses to the questions are shown in Figures 1 and 2, comparing the results of both rounds of the study. Participants were asked to rate the importance of having shared knowledge/beliefs for each of the mental model contents only once – either in the first round for the original set of contents *or* in the second round for newly added contents. In both rounds, the average ratings for importance were high: 75% and 84% respectively (percent strongly agree, defined as a score of 6 or 7 on a 7-point scale); hence these results are not included in the Figures. The ratings for the clarity, comprehensiveness and usefulness of the terms and definitions were lower than those for importance with, on average, only half of respondents providing a high rating (defined as a score of 6 or 7 on a 7-point scale); the results were similar for respondent views of how useful the framework is for interpreting, planning, implementing, managing and evaluating integration initiatives.

**Table 1:** Personal characteristics of respondents

**Characteristic**	**Category**	**Round I (n = 90)**	**Round II (n = 68)**
Gender	Female	**74.4%**	**72.1%**
	Male	25.6%	27.9%
Age	20-30	13.3%	14.7%
	31-45	26.7%	26.5%
	46-60	**45.6%**	**41.2%**
	61+	14.4%	17.6%
Region	Central Provinces	**76.7%***(73% ON)*	**83.8%***(79% ON)*
	Western Provinces	13.3%	8.8%
	Atlantic Provinces	6.7%	3.0%
	Prairie Provinces	3.3%	4.4%
Current Role^1^	Manager/Administrator	**56.7%**	**52.9%**
	Policymaker	17.8%	13.2%
	Researcher/Academic	14.4%	14.7%
	Consultant	14.4%	16.2%
	Clinician/Care Provider	12.2%	13.2%
	Patient/Caregiver Advocate	6.6%	5.9%
	Educator	5.6%	4.4%
Current Workplace^1^	Coordinating/Advisory Body	**19.0%**	**22.1%**
	Hospital	**19.0%**	14.7%
	Home & Community Care	16.7%	16.2%
	Ministry/Government	14.4%	11.8%
	RHA/DHA/LHIN^2^	12.4%	11.8%
	Primary Care	11.1%	14.7%
	University or Research Institute	11.1%	11.8%
	Professional Association/College	9.0%	8.8%
	Long-Term Care	2.2%	2.9%
Level of Integration^3^	Micro	23.3%	23.5%
(experience)	Meso	**47.8%**	**47.1%**
	Macro	28.9%	29.4%
Integration Activities^1^	Planning	**81.1%**	**73.5%**
(experience)	Implementation	64.4%	57.4%
	Management	57.8%	52.9%
	Evaluation	55.6%	52.9%
	Patient Care	35.6%	30.9%
	Policy-Making	31.1%	26.5%
	Research	29.0%	23.5%
	Patient/Caregiver Advocacy	14.4%	11.8%
	No Direct Experience	3.3%	4.4%

^1^Respondents could select multiple options.

^2^RHA/DHA: Regional/District Health Authority; LHIN: Local Health Integration Network.

^3^Macro: integration for entire communities; Meso: integration for specific patient or caregiver populations; Micro: integration for individual patients and their caregivers.

The main themes elicited from the review of open comments and the changes made in response to them are summarized in Table 2. Based on the quantitative and qualitative analysis, key findings included the need to: explain the framework’s purpose and potential applications, clarify some terms and definitions, modify the structure of the framework, and include additional content. The most important changes involved modifying the title of the framework from “Mental Models of Integration” to “Integration Mindsets” to enhance clarity and understanding, and the inclusion of eight new content areas in the beliefs/perceptions components of the framework to address gaps identified by the respondents. These additional content areas were identified using respondent comments (Table 3) and are supported by literature on health systems integration [e.g., 7,9,11].

**Table 2:** Results from thematic analysis of open comments

**Main themes**	**Modifications made**	**Comments**
**Round One**	**Round One**	**Round One**
Clarify the:	Provided a three-page overview to explain:	We retained the term “mental model” for two reasons: (a) no other term conveys the intended meaning and (b) the term maintains the link between this work and the literature on mental models.
• Purpose of the framework	• The value of a cognitive perspective on integration	
• Structure of the framework, particularly the section on beliefs/perceptions	• The broad meaning of the term “shared”	
	• What the framework aims, and does not aim, to do	
• Practical applications of the framework	• How the framework may be used, including examples	In the original framework, the beliefs/perceptions mental model consisted of the same contents as in the knowledge category, but with a focus on “what *should* be” or “what is *perceived* or *expected*”. For example, “clients” can be viewed in terms of which populations are being targeted for integrated care (knowledge of the integration strategy) versus which populations *should* be targeted (personal belief/perception). Repetition of the content areas created confusion and the contents failed to capture important beliefs/perceptions. Respondents also noted that their way of thinking about integration was complex and dynamic, incorporating knowledge, beliefs and perceptions; they therefore did not view beliefs/perceptions as a separate mental model.
• Terms and definitions in some parts of the framework, including the terms “mental model” and “shared”	Replaced the term “Mental Models of Integration” with “Integration Mindset”	
• Patient/caregiver perspective and role	Removed “belief/perceptions” as a separate type of mental model and added it as a component to the two remaining mental model types	
Include additional content, particularly in the area of beliefs/perceptions	Developed additional content to include in the framework, primarily on beliefs/perceptions:	
	• Strategy: “evaluation” added as a knowledge component and four beliefs/perceptions added	
	• Roles: four beliefs/perceptions added	
	Edited several terms and definitions in the framework for clarity and comprehensiveness. For example:	Additional framework contents identified through respondent comments and literature.
	• Strategy: “services to be integrated” changed to “targets”; “external customers changed to “clients”; and “processes” changed to “methods”	
	• Strategy: the definition for knowledge of clients was changed from “patients/caregivers who will benefit from integration” ***to*** “characteristics and needs of the populations, patients and/or caregivers who will benefit from integration, and the nature of that benefit”	
	• Roles: “knowledge and skills” changed to “competencies”; “role recognition” changed to “accountabilities”; and “interaction mechanisms” changed to “communication”. Minor modifications were made to the definitions for these terms as well (i.e. “role recognition: the purpose and responsibilities of each professional, organization and client” ***was replaced with*** “accountabilities: the activities and results that team members are individually or jointly responsible for”).	
**Round Two**	**Round Two**	**Round Two**
Reduce the number of content areas to improve clarity and reduce overlap	Replaced the term “Roles Mental Model” with “Relationships Mental Model”	Reducing the number of content areas may be premature without further research.
	Applied minor edits to some terms and definitions. For example:	
Further clarify some terms and definitions in the framework	• Strategy: “aptitude for change” changed to “readiness for change”	Several respondents noted that the term “role” focused attention on individual professionals and emphasized boundaries.
Further explain why factors such as culture and leadership are not included in the framework	• Relationships: we originally described the content for “each participating professional” and “each unit”, the latter defined as a program, department or organization. To improve clarity we now describe each content area using inter-professional and inter-organizational “team members” as the referent point.	The next stage of this research aims to develop a measurement tool for capturing and comparing Integration Mindsets as well as practitioner tools.
Develop an assessment tool or checklist to facilitate application	• Relationships: “recognition of shared responsibility: willingness to go beyond what one is obliged to do to support or contribute to the integration process” ***was replaced with*** “recognition of shared responsibility: a willingness to share the burden of work and act as a team to contribute to the integration process and/or to the delivery of integrated care”	
	Updated the three-page overview with an explanation of the range of contextual factors that influence (but are not inherently a part of) Integration Mindsets	

**Table 3:** Sample respondent comments used to identify beliefs/perceptions content

**Content**	**Sample respondent comments**
***Strategy Mental Model***	
Consequences of integrating	• “To be successful each participant must see benefit for him/her as well as the collective”
	• “Without willingness to accept and acknowledge the value of integration, it will be difficult to make progress”
Appropriateness of selected strategy	• “Need to include the concept of agreement with the goals, long-term vision and methods”
	• “Although there may be shared knowledge unless there is acceptance and commitment there will be challenges”
Integrity of decision-making processes	• “Decisions [must] have clear rationale that can be publicly defended”
	• “Important to have all views represented at the decision-making table”
Readiness for change	• “Add a concept about willingness to put energy into finding out, testing hypotheses, innovation”
	• “Include individual’s belief about their perceived freedom or ability to make change or execute integration activities”
***Relationships Mental Model***	
Appropriateness of role structure	• “It goes beyond knowledge to understanding, appreciation and agreement on the roles”
	• “Knowledge without buy-in is not sufficient”
Identification with the integration initiative	• “Each participant must have enthusiasm and investment in the initiative and must think beyond their current boundaries”
	• “Crucial to know why they are there: voluntarily or a directed (forced) integration?”
Recognition of shared responsibility	• “If the involved parties don’t recognize the need for specific organization engagement, the overall integration activity may not gain traction”
	• “More emphasis on interdependence, being more collaborative, recognizing that each brings expertise”
Importance of client involvement	• “The key issue is the integration of patient/family into interprofessional teamwork and this is dependent on changing current attitudes about practice and patient involvement”
	• “Most important players in planning and implementing integration are the persons and their families”

### Round II questionnaire

In the second round, 68 individuals responded (75.6% response rate). Over 95 comments were provided. In general, the ratings and comments were less critical than those provided in round one. Less than 3% of respondents gave a low rating (between 1 and 3 on a 7-point scale) for any of the survey items. The overwhelming majority of responses (85-96%) fell between 5 and 7 on a 7-point scale compared to only 63-82% in the first round, representing an average increase of 16%. The ratings increased substantially for the practical applications of the framework as a whole and for the Strategy Mental Model contents (Figures 1 and 2 respectively). For the Roles Mental Model (later renamed the Relationships Mental Model), the ratings only increased for comprehensiveness while views on clarity remained the same and views on usefulness decreased (Figure 1). This result suggests that there may be a trade-off between comprehensiveness and complexity, and the perceived usefulness of the contents.

The main themes elicited from the review of open comments and the changes made in response to them are summarized in Table 2. The quantitative and qualitative analysis identified the need to further clarify some terms and definitions as well as to reiterate the scope of the framework. Although ratings for comprehensiveness increased by about 20% in round two for both the Strategy and Roles Mental Model, upon analysis of respondent comments, it was apparent that most respondents were judging comprehensiveness based on the framework’s inclusion of influencing factors. Several respondents suggested that leadership, organizational culture, and policy be included in the framework. Although these are important factors that influence the implementation and success of integration initiatives, they describe the context for integration and not knowledge and beliefs regarding integration strategies.

### Cross-group agreement by level of integration experience, role, and workplace

Analysis of cross-group agreement revealed some statistically significant group-level differences in responses, primarily in ratings of usefulness of the concepts and framework (Additional file 3). Among the three respondent characteristics tested – level of integration experience, current role, and current workplace – only level of integration experience had a consistent relationship with group-level responses across both rounds of the study. In the first round, respondents with micro-level integration experience provided higher ratings than those with meso- and macro- level experience in select areas, primarily related to usefulness. In the second round, however, a significant difference between groups was detected for only one survey item which examined the usefulness of the framework for managing integration. Those with meso-level integration experience provided a lower mean rating (5.6 on a 7-point scale) for this question than those with micro- and macro-level experience (6.1 on a 7-point scale).

In regards to current role, respondents in a management, administration or consultant role initially provided higher ratings in comparison with other groups, particularly researchers, for the usefulness of the framework for interpreting, planning, implementing, managing, and evaluating integration. However, in the second round there were no significant differences identified in ratings across the four role types, which may suggest an increase in cross-group agreement. While ratings did not vary by respondents’ workplace in round one, they did in round two in relation to two survey items. For these two survey items, which examine usefulness of the “Roles Mental Model” concept and usefulness of the framework for interpreting integration experiences, ratings were generally lower among those working for a coordinating or advisory body, or for the government, than those working in other settings. This may be because individuals working in these settings are farther removed from direct patient care. For example, most of the coordinating and advisory bodies represented in the sample are focused on health system performance measurement and reporting.

In summary, the results suggest that respondents typically agreed on the clarity, comprehensiveness, and importance of the proposed concepts and framework; the key point of contention was in regards to how useful the concepts and framework are in practice and in what ways. Such differences are expected given the diversity of integration foci and methods, and the variety of professional groups and organizations involved in integration. Respondents who were “closer” to patients, such as managers and care providers working in healthcare settings, generally provided higher ratings than researchers, policymakers, or those working for advisory bodies. However, there were fewer differences detected in round two compared with round one, which may indicate increased cross-group agreement.

### Overview of the integration mindsets framework

The final version of the Integration Mindsets Framework is outlined in Table 4. An “Integration Mindset” refers to an individual’s way of thinking about integration that is based on knowledge and beliefs regarding the strategy for achieving integration (i.e., Strategy Mental Model) and the roles and relationships of those involved in the integration process (i.e., Relationships Mental Model). The contents of these two mental models, defined in Table 5, represent areas where a lack of shared knowledge and shared beliefs may negatively impact integration efforts. The framework is not intended to capture the process of integration or to reflect all of the factors that influence integration. The framework is also not designed as a prescriptive tool to “impose” strategies and “assign” roles. Rather, the framework is intended to aid discussion and measurement. The aims are to identify which knowledge and beliefs influence integration and to enhance understanding of the multiple conceptualizations of integration so that differences can be identified, unpacked, and explored; the desired outcome of this process is a synthesis of perspectives that is qualitatively better than any of the individuals’ perspectives. The framework is intentionally broad to permit its use for inter-organizational and inter-professional teams at macro, meso, or micro levels. The framework may also be adapted for use at different stages of integration.

**Table 4:** Integration mindsets framework

**Mental Model Type**	**Definition**	**Knowledge *(information & awareness)***	**Beliefs/Perceptions *(opinions or internal feelings)***
Strategy Mental Model	A conceptualization of what is being integrated and how, why and for whom it is being integrated	• Targets	• Consequences of integrating
		• External clients	• Appropriateness of selected strategy
		• Goals	• Integrity of decision-making processes
		• Long-term vision	• Readiness for change
		• Methods	
		• Evaluation	
Relationships Mental Model	A conceptualization of the organizations, groups, and individuals (including one’s self) involved in integration and how they are connected	• Competencies	• Appropriateness of role structure
		• Contributions	• Identification with the integration initiative
		• Accountabilities	• Recognition of shared responsibility
		• Interdependencies	• Importance of client involvement
		• Communication	

**Table 5:** Definitions of concepts in the integration mindsets framework

**Term**	**Definition**
**Strategy Mental Model – Knowledge Content**	
Targets	functions, services, organizations and/or systems identified for integration
External clients	characteristics and needs of the populations, patients and/or caregivers who will benefit from integration, and the nature of that benefit
Goals	primary aims of integration, which may be related to costs, efficiency, productivity, quality of care, patient safety and/or patient outcomes
Long-term vision	how the services, programs or functions, organizations and/or systems will “look” or operate when fully integrated
Methods	approaches and enablers for achieving integration – which may be clinical, technological, patient or caregiver-centered, administrative, financial, organizational, governance and/or policy-related – and timeline for implementation
Evaluation	key performance dimensions and indicators for assessment of the integration initiative
**Strategy Mental Model – Beliefs\Perceptions Content**	
Consequences of integrating	the expected outcomes (positive and/or negative) of integration for one’s self, for other participating individuals and organizations, for external clients, and for the healthcare system
Appropriateness of selected strategy	the extent of agreement with the selected targets, clients, goals, long-term vision, methods and evaluation approach for an integration initiative
Integrity of decision-making processes	the extent to which decisions regarding integration are made in a manner that is equitable and transparent
Readiness for change	the ability and willingness to implement the desired integration initiative
**Relationships Mental Model – Knowledge Content**	
Competencies	the knowledge and skill sets of each team member^1^
Contributions	how each team member contributes to patient health and well-being
Accountabilities	the activities and results that team members are individually or jointly responsible for
Interdependencies	how and to what extent the work of each team member depends on or is influenced by another
Communication	sources of information and how information flows between team members, including frequency and methods for contact
**Relationships Mental Model – Beliefs\Perceptions Content**	
Appropriateness of role structure	the extent of agreement with the content and distribution of roles, including relative accountabilities and communication methods
Identification with the integration initiative	the extent of self-association with the integration initiative (i.e. the team, partnership, network, etc.) in addition to one’s professional group and organization
Recognition of shared responsibility	a willingness to share the burden of work and act as a team to contribute to the integration process and/or to the delivery of integrated care
Importance of client involvement	the extent to which the involvement of patients and their caregivers is considered necessary and beneficial to integration efforts

^1^The term “team member” is used broadly to refer to individuals, organizations, and patients/caregivers participating in the integration initiative; the composition of the team will depend on the nature and level of the integration activity. These teams typically span professional and organizational boundaries, may be focused on governance, management or patient care, and may be formal or informal and ad hoc/intermittent or fixed.

## Discussion

The Integration Mindsets Framework aims to identify the cognitive contributors to integration success or failure. The framework outlines important knowledge and beliefs whose convergence or divergence across stakeholder groups may influence inter-professional and inter-organizational relations. Although SMMs are only one of many factors that influence integration, they may help to explain variations in performance, and may be leveraged to accelerate change in tandem with modifications to policy and legislation, organizational structures and context, and administrative and care processes.

A cognitive perspective on integration enhances our understanding of normative integration and facilitates its measurement, in part by complementing a cultural lens. Frameworks and methods used to measure “integrated system culture” provide limited, high-level insights [37] that are often not actionable, which may explain why integration evaluation methods rarely measure the cultural aspects of integrating [38]. The Integration Mindsets Framework allows us to explore and potentially measure the micro-foundations of culture with a focus on knowledge and beliefs *specific* to integration as opposed to general cultural attributes. Knowledge is of particular importance for two reasons: (1) the absence of knowledge in frameworks of organizational culture, and (2) knowledge of a change (i.e., what the change is, how it will impact the organization, how it will be implemented, etc.) has been linked to less resistance to change [39]. Questionnaires or interview guides may be developed to collect data on each of the content areas listed in Table 4 from various stakeholders involved in an integration activity, including managers and clinicians from organizations across the continuum of care such as hospitals, long-term care homes and community-based agencies. This type of data will complement the high-level information captured by cultural tools on an organization’s orientation in regards to people, innovation, control and outcomes [10], and provide a more complete understanding of normative integration in a given context and at a given point in time.

The two-round Delphi process allowed the original framework by Evans and Baker [20] to be tested against expert opinion. The study design created an opportunity for structured dialogue on integration among a varied group of healthcare stakeholders, and between investigators and participants. The respondents represented diverse policy contexts, professional roles, and organization types, thereby supporting the validity and generalizability of the concept of “Integration Mindsets” and the accompanying framework. Respondents asked questions, offered suggestions, and shared examples from their professional experiences; this rich feedback helped to clarify and further develop the concepts, structure and practical value of the framework while retaining and validating the underlying theoretical foundations. After the second round, there was strong interest among participants in the development of assessment or discussion tools for use in the field as well as vignettes or cases demonstrating use of the framework.

Many of the limitations associated with the Delphi method relate to small sample size and the identification, selection and commitment of the expert panel members [35]. In this study, we used a random sample of experts who attended a national symposium on integrated care, thereby reducing selection bias. Our sample was relatively large (n = 90) and response rates relatively high (52% and 76% respectively) in comparison to most Delphi and survey studies respectively [35,36]. Although respondents were overwhelmingly female (74%), from the province of Ontario (73%), and in a management position (57%), these distributions reflect the broader population of symposium attendees. While the diversity of the respondents and organizations represented support generalizability of the framework, further research is required to determine generalizability beyond the Canadian context. In addition, patient and caregiver perspectives were not captured because the framework and method of study would require modification to appropriately reflect and elicit their views; however, the framework does conceptualize patients and caregivers as valued members of the healthcare team, and patient advocates did participate in the study (n = 6). Finally, although the Kruskall-Wallis test is robust in detecting differences across groups with small sample sizes (n ≥ 5), the sub-group sample sizes by respondent level of integration, workplace and role were unequal with relatively large ranges (e.g., fifty-three managers versus eleven researchers). The results must therefore be interpreted with caution.

## Conclusion

Given the cultural and cognitive challenges to integration [6-9,40,41], there is an increasing need for research and practical tools that can enhance our understanding and ability to measure and influence normative integration. The Integration Mindsets Framework offers a starting point for doing so by drawing our attention to how various stakeholders involved in a specific integration initiative think about and conceptualize integration. The cognitive and cross-boundary (i.e., inter-organizational and inter-professional) focus of the framework supports recommendations from the change management literature to engage individuals and groups at all levels in leading and participating in change efforts, to pay attention to change recipients’ emotional, cognitive, and behavioral reactions to change, and to address divergent visions and goals or mis-alignments in the knowledge and beliefs needed for the change [42-44].

We can use the Integration Mindsets Framework to explore: How do mindsets evolve (or not) with implementation? How do views differ among leaders and staff, providers and managers, and providers and patients/caregivers? How similar are mindsets among different organizations involved in integration? These questions may be explored through discussions among team members or partnering organizations using the framework as a guide, or through formal measurement once a measurement tool has been developed for capturing and comparing integration mindsets. Below we offer more specific examples of potential applications of the framework.

•**Interpretation:** Current or past integration efforts may be (re-)interpreted using the framework. For example, despite careful redesign of structures and processes, and a favourable environment, some integration efforts still fail to meet objectives. Differences in integration mindsets may help partly explain such cases.

•**Planning:** The framework can be used to direct and focus early discussions and planning efforts among team members or partnering organizations, and to assess system or organizational readiness for integration.

•**Implementation:** The framework draws our attention to important knowledge content, some of which may be co-created or clarified, recorded, and formally agreed to during the integration implementation stage.

•**Management:** Awareness of the extent to which integration mindsets are shared and where similarities and differences lie can help guide change management interventions. A lack of cross-understanding (possessing an accurate understanding of the mental models of others) negatively impacts collective learning and performance [45]. Managers and leaders can use information on integration mindsets to identify pockets of resistance to change or to leverage the support of champions, to re-frame the initiative, as well as to develop interventions which align with the identified problem [46,47]. With regards to the latter, for example, a lack of shared knowledge may be addressed through training and education, whereas a lack of shared beliefs may require more extensive and potentially long-term dialogue and negotiation in addition to changes to structures and incentives [20].

•**Evaluation:** The extent to which integration mindsets are shared may help assess the success and sustainability of an integration activity. As relationships develop and new work practices become embedded over time, actors retrospectively make sense of what they did together and knowledge and beliefs/perceptions become more congruent [48,49]. “Shared integration mindsets” may be used as one indicator, among many, of a successful and sustainable integration activity.

In addition to improving the Integration Mindsets Framework in preparation for empirical research and practical use, this study also establishes a common language for discourse on cognition in health systems integration and encourages interdisciplinary and cross-level theorizing. Further research is needed to determine the contribution of the Integration Mindsets Framework to research and practice. In particular, future studies should examine the framework’s relevance in various countries and health systems, and develop and test measurement tools for capturing and comparing “integration mindsets” with attention to psychometric properties. An international Delphi panel is an appropriate next step to examine the framework’s generalizability beyond the Canadian context and to further validate the framework prior to its application. Incorporating patient views is also fundamental to the validation process. Complex patients accustomed to interacting with multiple care providers may be briefed on the structures and processes associated with a particular integrated care model, and asked what they think their providers need to agree on – and what shared knowledge and beliefs they require – to work together effectively under that model. This open-ended discussion may be followed by a more focused discussion of the contents of the framework and the extent to which the contents resonate with patients. Future research will also help clarify the nature of the framework’s contents and their relative importance to collaboration. For example, with regards to the “evaluation” component of the Strategy Mental Model, is having a shared awareness of what the metrics are important in and of itself? Or is having a shared belief in the validity of the metrics and individual or collective ability to influence the metrics important? In addition, how might the influence of convergent and divergent integration mindsets differ based on the type and level of integration, the degree of similarity or dissimilarity, the number and diversity of actors involved, and the context? In summary, a better understanding of the evolution and interplay of meanings, interpretations and knowledge about integration across inter-professional and inter-organizational boundaries may help to accelerate progress towards integrated care.

## Competing interests

The authors declare that they have no competing interests.

## Authors’ contributions

JME conceived of the study, conducted the data collection and data analysis, and drafted the manuscript. GRB, WB and JB participated in the design of the study and provided critical commentary on the manuscript. All authors read and approved the final manuscript.

## Pre-publication history

The pre-publication history for this paper can be accessed here:

http://www.biomedcentral.com/1472-6963/14/222/prepub

## Supplementary Material

Additional file 1 Round I Questionnaire. Click here for file

Additional file 2 Round II Questionnaire. Click here for file

Additional file 3 Summary of Sub-Group Differences by Respondents’ Level of Integration Experience, Current Workplace and Current Role. Click here for file
