# Supplementary material for: A cognitive perspective on health systems integration: results of a Canadian Delphi study
Source: BMC Health Serv Res. 2014 May 19;14:222. doi: 10.1186/1472-6963-14-222 (PMC4066828; doi:10.1186/1472-6963-14-222)
Supplement: Additional file 1 — Round I Questionnaire. [file 1472-6963-14-222-S1.pdf]

Round I Questionnaire

INTRODUCTION

Health systems around the world are implementing integration strategies to improve the efficiency and quality of health care services. Integration involves bringing organizations, services, and professionals together with the aim of improving outcomes for patients through the delivery of integrated care.

This study seeks to explore the potential contributions of a psychological perspective on integration through the development of a framework of "Mental Models of Integration" (MMI). A *mental model* is a way of thinking about how something works. MMI are thus ways of thinking about integration activities in the health care sector.

In this questionnaire, we are interested in your assessment of our framework of MMI, which we describe on page 2.

DIRECTIONS

In the pages that follow, we provide you with a brief overview of "Mental Models of Integration" (MMI) and a questionnaire consisting of three sections.

1. Assessment of the Framework
2. Significance of the Framework
3. Participant Information

The questionnaire will take approximately 30 minutes to complete.

Please submit your completed questionnaire by Tuesday November 6th 2012.

You have two submission options:

1. Complete this word document and send it to Jenna.Evans@utoronto.ca **OR**
2. Complete on the Web at https://www.surveymonkey.com/s/MMIntegration.

Thank you for taking the time to complete this questionnaire.
Your response is very important to us.

OVERVIEW OF MENTAL MODELS OF INTEGRATION

Mental Models of Integration (MMI) are **ways of thinking about integration activities in the health care sector.** These mental models are based on knowledge and beliefs relevant to integration, and are used by individuals to guide analysis and action. Knowledge refers to information and awareness about an integration activity, while beliefs refer to preferences and expectations regarding an integration activity.

Individuals, including clinicians, managers, administrators, board members and policymakers, develop and refine their MMI through their education and training as well as through personal and professional experiences or observations of integration.

Our review of theory and evidence suggests that it is important for individuals and organizations working together on an integration activity to have **shared** MMI in three areas: **strategy, roles, and beliefs**. The definition and contents of each of these mental model categories are outlined in the table below.

MMI	Definition	Content
Strategy	way of thinking about integration based on information and awareness (i.e. knowledge) about what is being integrated and how, why and for whom it is being integrated	▪ Targets<br>▪ Clients<br>▪ Goals<br>▪ Long-term vision<br>▪ Methods
Roles	way of thinking about integration based on information and awareness (i.e. knowledge) about the individuals, groups, and organizations involved in integration, and their interactions	▪ Knowledge and skills<br>▪ Role recognition<br>▪ Role contribution<br>▪ Role interdependence<br>▪ Interaction patterns
Beliefs	way of thinking about integration based on preferences or expectations	All of the above, but with a focus on preferences and expectations as opposed to information and awareness

Information about the views of individuals and teams in the areas listed above may offer new insights into factors that support or prevent further integration of services. This information can complement existing measures and change management efforts, and help us better understand cultural differences and conflicts. Practical methods, such as survey and discussion tools based on our MMI framework, can also be developed to inform integration planning and evaluation, or to guide inter-organizational and inter-personal dialogue.

Further details on our MMI framework are provided throughout the questionnaire. In addition, optional background reading is provided on page 18 should you desire more information.

1. Please enter your e-mail address:

[ ]

2. Which of the following levels of integration best describes your experience in integration activities? Although these three levels are often used in combination, please select only ONE level.

☐ **Macro** (integration for entire communities irrespective of health status; the focus is on integrating organizations and their activities, and/or modifying governance, finance, policy, and regulatory mechanisms)

Example: Local Health Integration Networks in Ontario

☐ **Meso** (integration for patient populations with the same disease or condition or for particular groups of caregivers; the focus is on integrating organizations and their activities, and/or integrating health and social services)

Example: integrated service program for the frail elderly

☐ **Micro** (integration for individual patients and their caregivers; the focus is on integrating health and social services at the point of care)

Example: inter-disciplinary teamwork and case management

In assessing our framework of "Mental Models of Integration" in the pages that follow, it may be helpful to keep in mind the level of integration you selected here.

SECTION I: ASSESSMENT OF THE FRAMEWORK

In this section, we are interested in determining how **clear**, **comprehensive**, and **useful** the terms, definitions, and contents of the framework are from your perspective. We are also interested in your assessment of the **importance** of the contents.

We will begin by asking for your feedback on the first category of "Mental Models of Integration" (MMI): Strategy. Please review the information below before answering the questions.

Term	Definition
Strategy MMI	way of thinking about integration based on information and awareness about what is being integrated and how, why and for whom it is being integrated

This mental model is related to integration strategy, and is comprised of the following contents: targets, clients, goals, long-term vision, and methods. We suggest that **shared knowledge** (i.e. information and awareness) among individuals and teams in these five areas supports inter-professional and inter-organizational collaboration.

Term	Definition
Targets	services, programs or functions, and/or organizations identified for integration
Clients	patients/caregivers who will benefit from integration
Goals	primary objectives of integration, which may be related to costs, efficiency, quality of care, and/or patient outcomes
Long-term vision	how the services, programs or functions, and/or organizations will "look" or operate when fully integrated
Methods	approaches for achieving integration, which may be clinical, technological, patient or caregiver-centered, administrative, organizational, governance and/or policy-related

METHOD	EXAMPLE
Clinical	inter-disciplinary teamwork
Technological	shared electronic patient records
Patient or caregiver-centered	self-management program
Administrative	joint purchasing
Organizational	consolidation or strategic alliance
Governance	board member rotation
Policy	population, needs-based funding

3. How <u>clear</u> is the description of a "Strategy Mental Model" to you?

☐	☐	☐	☐	☐	☐	☐
1	2	3	4	5	6	7
**Not Clear**			**Somewhat Clear**			**Very Clear**

4. How <u>comprehensive</u> or complete are the contents of a "Strategy Mental Model" to you?

☐	☐	☐	☐	☐	☐	☐
1	2	3	4	5	6	7
**Not Comprehensive**			**Somewhat Comprehensive**			**Very Comprehensive**

4. To what extent is the concept of a "Strategy Mental Model" <u>useful</u> to you in considering integration efforts and experiences?

☐	☐	☐	☐	☐	☐	☐
1	2	3	4	5	6	7
**Not Useful**			**Somewhat Useful**			**Very Useful**

6. Please provide comments or suggestions on the <u>clarity</u>, <u>comprehensiveness</u> and/or <u>usefulness</u> of the concept of a "Strategy Mental Model of Integration". You may suggest alternative terms or definitions, or the removal or addition of content areas.

Based on your experiences, how important do you think it is for those involved in an integration activity to have shared knowledge (i.e. information and awareness) in each of the following areas of a "Strategy Mental Model"?

	Not Important			Somewhat Important			Very Important
**7. Targets** (services, programs or functions, and/or organizations identified for integration)	☐ 1	☐ 2	☐ 3	☐ 4	☐ 5	☐ 6	☐ 7
**8. Clients** (patients/caregivers who will benefit from integration)	☐ 1	☐ 2	☐ 3	☐ 4	☐ 5	☐ 6	☐ 7
**9. Goals** (primary objectives of integration, which may be related to costs, efficiency, quality of care, and/or patient outcomes)	☐ 1	☐ 2	☐ 3	☐ 4	☐ 5	☐ 6	☐ 7
**10. Long-term vision** (how the services, programs or functions, and/or organizations will "look" or operate when fully integrated)	☐ 1	☐ 2	☐ 3	☐ 4	☐ 5	☐ 6	☐ 7
**11. Methods** (approaches for achieving integration, which may be clinical, technological, patient or caregiver-centered, administrative, organizational, governance, and/or policy-related)	☐ 1	☐ 2	☐ 3	☐ 4	☐ 5	☐ 6	☐ 7

12. Please provide comments or suggestions on the <u>importance</u> of having a <u>shared</u> "Strategy Mental Model of Integration". You may also comment on the importance of other content areas not listed above.

Now we will ask for your feedback on the second category of "Mental Models of Integration" (MMI): <u>Roles</u>. Please review the information below before answering the questions.

Term	Definition
Roles MMI	way of thinking about integration based on information and awareness about the individuals, groups, and organizations involved in integration, and their interactions

This mental model is related to integration roles, and is comprised of the following contents: knowledge and skills; role recognition; role contribution; role interdependence; and interaction mechanisms. We suggest that **<u>shared knowledge</u>** (i.e. information and awareness) among individuals and teams in these five areas supports inter-professional and inter-organizational collaboration.

Term	Definition
Knowledge and skills	the capabilities and expertise of each professional, organization, and client
Role recognition	the purpose and responsibilities of each professional, organization, and client
Role contribution	how each role (professional, organizational, client) contributes to patient health and well-being
Role interdependence	how and to what extent each role (professional, organizational, client) depends on, is influenced by, or is accountable to another
Interaction mechanisms	sources of information, how information flows, particularly at professional and organizational hand-offs, and the frequency and mechanisms for contact

13. How <u>clear</u> is the description of a "Roles Mental Model" to you?

☐	☐	☐	☐	☐	☐	☐
1	2	3	4	5	6	7
**Not Clear**			**Somewhat Clear**			**Very Clear**

14. How <u>comprehensive</u> or complete are the contents of a "Roles Mental Model" to you?

☐	☐	☐	☐	☐	☐	☐
1	2	3	4	5	6	7
**Not Comprehensive**			**Somewhat Comprehensive**			**Very Comprehensive**

15. To what extent is the concept of a "Roles Mental Model" <u>useful</u> to you in considering integration efforts and experiences?

☐	☐	☐	☐	☐	☐	☐
1	2	3	4	5	6	7
**Not Useful**			**Somewhat Useful**			**Very Useful**

16. Please provide comments or suggestions on the <u>clarity</u>, <u>comprehensiveness</u> and/or <u>usefulness</u> of a "Roles Mental Model of Integration". You may suggest alternative terms or definitions, or the removal or addition of content areas.

Based on your experiences, how <u>important</u> do you think it is for those involved in an integration activity to have <u>shared knowledge</u> (i.e. information and awareness) in each of the following areas of a "Roles Mental Model of Integration"?

	Not Important			Somewhat Important			Very Important
**17. Knowledge & skills** (the capabilities and expertise of each professional, organization, and client)	☐ 1	☐ 2	☐ 3	☐ 4	☐ 5	☐ 6	☐ 7
**18. Role recognition** (the purpose and responsibilities of each professional, organization, and client)	☐ 1	☐ 2	☐ 3	☐ 4	☐ 5	☐ 6	☐ 7
**19. Role contribution** (how each role – professional, organizational, and client – contributes to patient health and well-being)	☐ 1	☐ 2	☐ 3	☐ 4	☐ 5	☐ 6	☐ 7
**20. Role interdependence** (how and to what extent each role – professional, organizational, and client – depends on, is influenced by, or is accountable to another)	☐ 1	☐ 2	☐ 3	☐ 4	☐ 5	☐ 6	☐ 7
**21. Interaction mechanisms** (sources of information, how information flows, particularly at professional and organizational hand-offs, and the frequency and mechanisms for contact)	☐ 1	☐ 2	☐ 3	☐ 4	☐ 5	☐ 6	☐ 7

22. Please provide comments or suggestions on the <u>importance</u> of having a <u>shared</u> "Roles Mental Model of Integration". You may also comment on the importance of other content areas not listed above.

Now we will ask for your feedback on the last category of "Mental Models of Integration" (MMI): <u>Beliefs</u>. Please review the information below before answering the questions.

Term	Definition
Beliefs MMI	way of thinking about integration based on preferences and expectations regarding integration

This mental model is related to integration beliefs, and is comprised of the content listed in the table below. You will notice that what distinguishes a Beliefs MMI from a Strategy or Roles MMI is an emphasis on "what *should* be" or "what is *perceived* or *expected*". These preferences and expectations (i.e. beliefs) are shaped by various factors, including education, training, personal or professional experiences, history, political interests, power differences, and accountability structures. For example:

- a board member may *know* that services for the elderly are being integrated, but may *believe* that services for all chronic disease patients should be integrated regardless of age;
- a clinician may be *aware* of the importance of linking patients to community-based resources, but may not *believe* that doing so is part of his/her role; and
- a senior manager may *believe* that the long-term vision of an integration activity is a take-over, even though the *true* intention is a collaborative partnership.

We suggest that **<u>shared beliefs</u>** (i.e. preferences and expectations) among individuals and teams in the ten areas below support inter-professional and inter-organizational collaboration.

Term	Definition
Targets	services, programs or functions, and/or organizations that **<u>should</u>** be integrated
Clients	patients/caregivers who **<u>should</u>** benefit from integration
Goals	objectives that **<u>should</u>** be driving integration, which may be related to costs, efficiency, quality of care, and/or patient outcomes
Long-term vision	how the services, programs or functions, and/or organizations **<u>should</u>** "look" or operate when fully integrated

(continued)

Methods	approaches that **<u>should</u>** be used for integration, which may be clinical, technological, patient or caregiver-centered, administrative, organizational, governance, and/or policy-related
Knowledge and skills	**<u>perceived or expected</u>** capabilities and expertise of each professional, organization, and client
Role recognition	**<u>perceived or expected</u>** purpose and responsibilities of each professional, organization, and client
Role contribution	**<u>perceived or expected</u>** way(s) in which each role (professional, organizational, client) contributes to patient health and well-being
Role interdependence	**<u>perceived or expected</u>** way(s) in which each role (professional, organizational, client) depends on, is influenced by, or is accountable to another
Interaction mechanisms	**<u>perceived or expected</u>** sources and flow of information, particularly at professional and organizational hand-offs, and **<u>perceived or expected</u>** frequency and mechanisms for contact

23. How <u>clear</u> is the description of a "Beliefs Mental Model" to you?

☐ 1	☐ 2	☐ 3	☐ 4	☐ 5	☐ 6	☐ 7
**Not Clear**			**Somewhat Clear**			**Very Clear**

24. How <u>comprehensive</u> or complete are the contents of a "Beliefs Mental Model" to you?

☐ 1	☐ 2	☐ 3	☐ 4	☐ 5	☐ 6	☐ 7
**Not Comprehensive**			**Somewhat Comprehensive**			**Very Comprehensive**

25. To what extent is the concept of a "Beliefs Mental Model" useful to you in considering integration efforts and experiences?

☐	☐	☐	☐	☐	☐	☐
1	2	3	4	5	6	7
**Not Useful**			**Somewhat Useful**			**Very Useful**

26. Please provide comments or suggestions on the clarity, comprehensiveness and/or usefulness of a "Beliefs Mental Model of Integration". You may suggest alternative terms or definitions, or the removal or addition of content areas.

Based on your experiences, how important do you think it is for those involved in an integration activity to have shared strategy-related beliefs and shared roles-related beliefs?

	**Not Important**			**Somewhat Important**			**Very Important**
**27. Strategy-related beliefs** (preferences and expectations regarding targets, clients, goals, long-term vision, and methods)	☐ 1	☐ 2	☐ 3	☐ 4	☐ 5	☐ 6	☐ 7
**28. Roles-related beliefs** (preferences and expectations regarding knowledge and skills, role recognition, role contribution, role interdependence, and interaction mechanisms)	☐ 1	☐ 2	☐ 3	☐ 4	☐ 5	☐ 6	☐ 7

29. Please provide comments or suggestions on the importance of having a shared "Beliefs Mental Model of Integration". You may also comment on the importance of other content areas not listed above.

Now we would like your feedback on the overarching concept of "Mental Models of Integration". Please review the information below and consider the content covered thus far to answer the questions.

Term	Definition
Mental Models of Integration	ways of thinking about integration activities in the health care sector, based on relevant knowledge and beliefs

30. How clear is this term and definition to you?

☐ 1	☐ 2	☐ 3	☐ 4	☐ 5	☐ 6	☐ 7
**Not Clear**			**Somewhat Clear**			**Very Clear**

31. How comprehensive or complete are the contents of "Mental Models of Integration" (Strategy, Roles, Beliefs) **to you in terms of identifying important areas where shared knowledge and beliefs are needed among those involved in an integration activity?**

☐ 1	☐ 2	☐ 3	☐ 4	☐ 5	☐ 6	☐ 7
**Not Comprehensive**			**Somewhat Comprehensive**			**Very Comprehensive**

32. Please provide comments or suggestions on the clarity and/or comprehensiveness of "Mental Models of Integration". You may suggest an alternative term or definition, or the removal or addition of mental model categories.

SECTION II: SIGNIFICANCE OF THE FRAMEWORK

In this section, we are interested in your assessment of the significance of the framework of "Mental Models of Integration" (MMI).

Please rate the extent to which you think that the MMI framework is useful for interpreting integration experiences as well as for planning, implementing, managing, and evaluating integration activities.

	Not Useful			Somewhat Useful			Very Useful
**33. Interpreting integration experiences**	☐ 1	☐ 2	☐ 3	☐ 4	☐ 5	☐ 6	☐ 7
**34. Planning integration activities**	☐ 1	☐ 2	☐ 3	☐ 4	☐ 5	☐ 6	☐ 7
**35. Implementing integration activities**	☐ 1	☐ 2	☐ 3	☐ 4	☐ 5	☐ 6	☐ 7
**36. Managing integration activities**	☐ 1	☐ 2	☐ 3	☐ 4	☐ 5	☐ 6	☐ 7
**37. Evaluating integration activities**	☐ 1	☐ 2	☐ 3	☐ 4	☐ 5	☐ 6	☐ 7

38. Please provide us with any comments or suggestions you may have regarding the <u>significance</u> of the MMI framework and its contents.

SECTION III: PARTICIPANT INFORMATION

In this final section, we are interested in learning more about your background.

39. Age: ☐ 20 – 30 ☐ 31 – 45 ☐ 46 – 60 ☐ 61+

40. Sex: ☐ Male ☐ Female

41. Province or Territory:

☐ AB	☐ NB	☐ NT	☐ PE	☐ YT
☐ BC	☐ NL	☐ NU	☐ QC	
☐ MB	☐ NS	☐ ON	☐ SK	

42. Which of the following best describes your <u>workplace</u>?

☐ Hospital
☐ Primary care clinic
☐ Long-term care home
☐ Home and community support agency
☐ Ministry/government
☐ RHA/LHIN
☐ Other coordinating and/or advisory body
☐ Professional association or college
☐ University/research institute
☐ Other – please specify: ____________

43. Which of the following best describes your <u>current role</u>? Check all that apply.

☐ Clinician/Care Provider
☐ Manager/Administrator
☐ Researcher/Academic
☐ Educator
☐ Policymaker
☐ Patient/Caregiver advocate
☐ Consultant
☐ Other – please specify: ____________

44. How <u>long</u> have you been working in your current role? [ ] year(s)

45. Which of the following best describes your <u>education/training</u>? Check all that apply and provide details in the spaces provided.

☐ Bachelor's Degree: [ ]
☐ Master's Degree: [ ]
☐ MD: [ ]
☐ PhD: [ ]
☐ Other – please specify: [ ]

46. Which of the following best describes your <u>experience</u> in integration activities? Check all that apply.

☐ Planning
☐ Implementation
☐ Management
☐ Patient care
☐ Evaluation
☐ Research
☐ Policy-making
☐ Patient/caregiver advocacy
☐ Other – please specify: [ ]
☐ No direct experience

<u>Thank you</u> very much for taking the time to complete this questionnaire.

OPTIONAL BACKGROUND READING

Most scholars and practitioners have focused on the structural and process aspects of integration, but collaboration across professional and organizational boundaries also requires attention to culture and cognition (i.e., mental models). While organizational and professional "culture" refers to dominant beliefs and behaviours, mental models are internal ways of thinking about specific tasks or situations. When multiple individuals develop a common psychological understanding of a task or situation, this is referred to as a "shared mental model". A "shared mental models" perspective on integration may help us better understand cultural differences and conflicts, while also offering new insights into the barriers and enablers to integration. We define "shared" as *overlapping*, not *identical*, ways of thinking about integration; perspectives will vary depending on roles and organizational context. However, a broad level of consensus allows for diversity while also providing the common meaning needed for collaboration.

Our MMI framework draws from research on shared mental models, systems thinking, and strategic management and change. Below we offer excerpts from the abstracts of four academic papers, among many, that have informed our framework.

Cannon-Bowers J, Salas E (2001) Reflections on shared cognition. ***Journal of Organizational Behavior*****, 22: 195-202.** The purpose of this paper is to highlight several fundamental questions...regarding shared cognition: What must be 'shared'? What does 'shared' mean? How should 'shared' be measured? What outcomes do we expect shared cognition to affect? A general and integrative description of these questions is provided...[and] the value of shared cognition is discussed...

Fiol C (1994) Consensus, Diversity and Learning in Organizations. ***Organization Science*****, 5: 403-420.** Organizational learning...involves the development of new and diverse interpretations of events and situations...collective learning also involves developing enough consensus around those diverse interpretations for organized action to result...People may hold very different pictures of reality and still agree on the way they frame them...managers must [strive] for a shared framing of the issues that is broad enough to encompass those differences.

Hysong S, et al (2005) Not of One Mind: Mental Models of Clinical Practice Guidelines in the Veterans Health Administration. ***Health Services Research*****, 40(3): 829-848.** The purpose of this paper is to present differences in mental models of clinical practice guidelines among 15 Veterans Health Administration facilities...We conclude that a clear shared mental model of guidelines, in combination with a learning orientation toward feedback are important components...

Vlaar P, et al (2006) Coping with Problems of Understanding in Inter-Organizational Relationships: Using Formalization as Means to Make Sense. ***Organization Studies*****, 27(11): 1617.** ...partners cooperating in such [inter-organizational] relationships are also confronted with 'problems of understanding'. Such problems arise from differences between partners in terms of culture [and] experience...and from the...ambiguity that participants...experience in...collaboration... the mechanisms through which formalization facilitates sensemaking [include]: (1) focusing participants' attention; (2) provoking articulation, deliberation and reflection; (3) instigating and maintaining interaction; and (4) ...diminishing the incompleteness and inconsistency of cognitive representations.
