# Supplementary material for: A cognitive perspective on health systems integration: results of a Canadian Delphi study
Source: BMC Health Serv Res. 2014 May 19;14:222. doi: 10.1186/1472-6963-14-222 (PMC4066828; doi:10.1186/1472-6963-14-222)
Supplement: Additional file 2 — Round II Questionnaire. [file 1472-6963-14-222-S2.pdf]

Round II Questionnaire

INTRODUCTION

In this questionnaire, we summarize the results from the first round and describe the revised framework, which incorporates your feedback. The questionnaire will take approximately 20-30 minutes to complete.

We sincerely appreciate your time and participation. Your response will greatly enhance expert dialogue and the study's results.

SUMMARY OF FEEDBACK FROM ROUND ONE

A total of 90 individuals responded from nine provinces across Canada (52% response rate). The participants consisted of managers and administrators (57%), policy-makers (18%), researchers (14%), consultants (14%), clinicians and care providers (12%), patient and caregiver advocates (7%), and educators (6%).[1]

Organizations from across the continuum of care were represented – including acute care (19%), home and community care (17%), primary care (11%), and long-term care (2%) – as well as government (14%), professional associations (9%), and a variety of coordinating or advisory bodies (31%).[1]

Of those that responded, 81% had experience in planning integration activities; 64% in implementation; 58% in management; 56% in evaluation; and 36% in patient care.[1]

On average,

- 75% rated shared mental models of integration as very important;
- 51% rated the concepts in the framework as very clear, very comprehensive, and very useful; &
- 51% rated the framework as very useful for planning and evaluating integration efforts.[2]

We reviewed and considered all of the ratings and the 250+ comments provided. Based on the quantitative and qualitative analysis, the main issues identified were the need to:

- explain the purpose and practical applications of the framework;
- situate the framework in relation to what we already know about the integration process;
- clarify some concepts and the structure of the framework, particularly the section on beliefs.

[1] Participants could select multiple options.
[2] Defined as a score of 6 or 7.

OVERVIEW OF THE INTEGRATION MINDSETS FRAMEWORK

Background: Why Focus on Integration Mindsets?

The figure below describes various types of integration in the health and social sectors. We are making progress in understanding, effecting, and measuring organizational, functional, service, and clinical integration. However, we still know little about *normative* integration. One way to explore normative integration is by examining organizational and professional cultures. Another way is to examine “integration mindsets”. We need both of these perspectives to better understand normative integration.

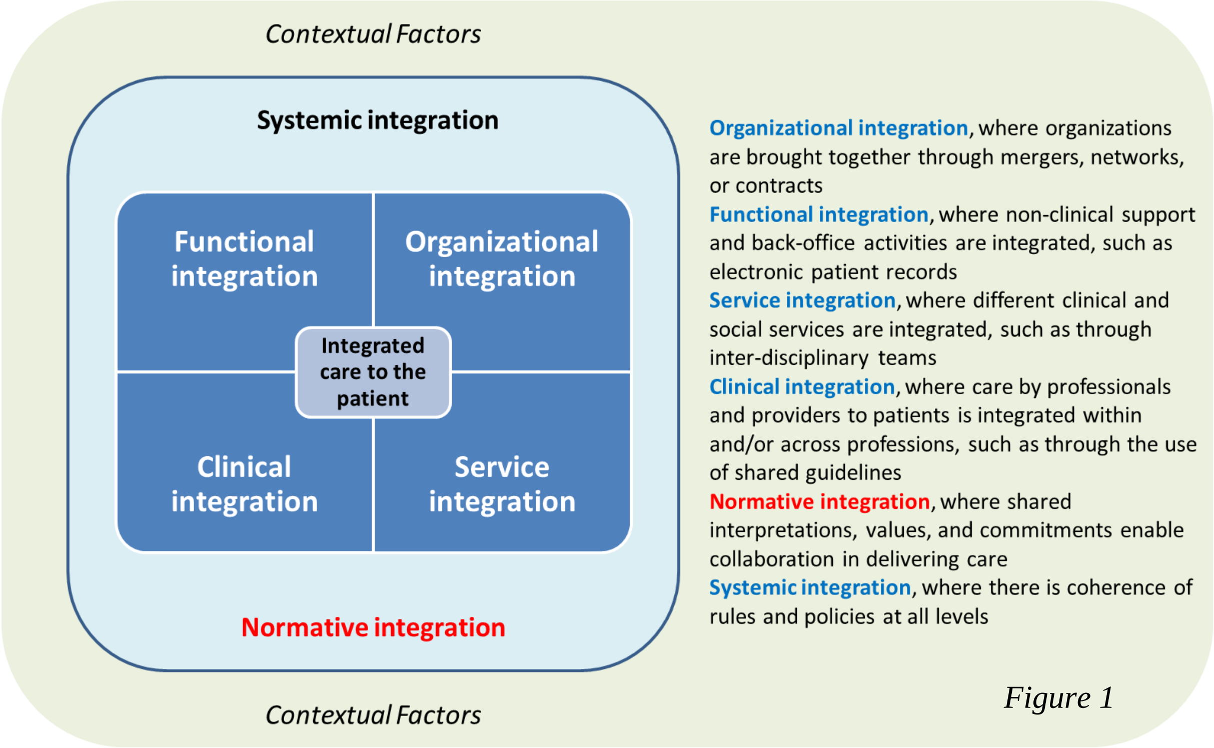

Figure 1

Source: Ham C & Curry N (2010) Clinical and service integration: The route to improved outcomes. The King’s Fund.

What is an Integration Mindset?

An **Integration Mindset** refers to an individual’s way of thinking about integration that is based on knowledge and beliefs regarding the strategy for achieving integration (i.e. strategy mental model) and the roles of those involved in the integration process (i.e. roles mental model).

The figure below outlines the expected benefits of <u>shared</u> integration mindsets. “Shared” is defined as a *similar* and *overlapping*, not an identical, mindset among a group of individuals.

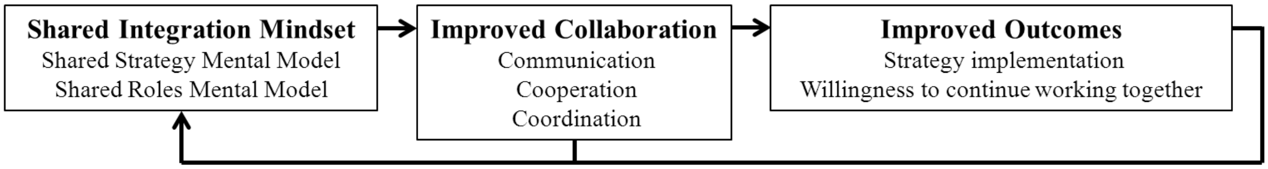

Figure 2

Framework of Integration Mindsets (revised based on round one) [1]

Table 1

Mental Model Type	Definition	Knowledge* (information & awareness)	Beliefs/Perceptions* (opinions or internal feelings)
Strategy Mental Model	a conceptualization of what is being integrated and how, why and for whom it is being integrated	▪ Targets<br>▪ Clients<br>▪ Goals<br>▪ Long-term vision<br>▪ Methods<br>▪ Evaluation	▪ Consequences of integrating<br>▪ Appropriateness of selected strategy<br>▪ Integrity of decision-making processes<br>▪ Aptitude for change
Roles Mental Model	a conceptualization of the individuals, groups, and organizations involved in integration, and their relationships	▪ Competencies<br>▪ Contributions<br>▪ Accountabilities<br>▪ Interdependencies<br>▪ Interactions<br>▪ Role impact	▪ Appropriateness of role structure<br>▪ Viability of hybrid identity<br>▪ Recognition of shared responsibility<br>▪ Importance of client involvement

[1] A table of essential background terms and their definitions is provided in the Appendix on page 15.
* These are areas where a lack of <u>shared</u> knowledge and <u>shared</u> beliefs may negatively impact integration efforts.

Key Points Based on Feedback from Round One

- The framework is not intended to capture the process of integration or to reflect all of the factors that influence integration. As shown in Figure 1, normative integration is only one aspect of integration.
- The framework is intentionally broad to allow for the concepts to be applied to integration initiatives involving inter-organizational and inter-professional teams at macro, meso, or micro levels. The framework may also be adapted for use at different stages of integration.
- The framework depicts patients and caregivers as valued team members.
- The framework is intended for measurement and discussion purposes, not for use as a prescriptive tool to "impose" strategies and "assign" roles.
- Conceptualizations of integration are shaped by the very process of working together. Education, dialogue, and leadership, among other factors, play a role in aligning mindsets.

How to Apply the Framework

- The framework draws our attention to how various stakeholders involved in a specific integration initiative <u>think</u> about and <u>conceptualize</u> integration. We can examine: How did mindsets and their degree of similarity evolve (or not) with implementation? How do these views differ between leaders and staff, providers and managers, and providers and patients/caregivers? These questions may be explored through discussions among team members or partnering organizations using the framework as a guide, or through formal measurement once a measurement tool has been developed.
- The resulting information can be used alongside other data to help (a) guide education and training, and change management efforts; (b) assess system or organizational readiness for integration; or (c) evaluate the ongoing success and sustainability of an integration initiative.

1. Please enter your e-mail address:

☐

2. To receive your complimentary <u>$10 coffee card</u>, please select your preferred brand and provide a mailing address.

☐ Starbucks ☐ Second Cup ☐ Tim Hortons

Mailing address:

☐

<u>SECTION I: Are the Concepts Clear & Comprehensive?</u>

Summary of Feedback from Round One

Average ratings for the clarity and comprehensiveness of the key concepts in the framework:

2%	3%	4%	16%	24%	36%	15%
1	2	3	4	5	6	7
**Not Clear & Comprehensive**			**Somewhat Clear & Comprehensive**			**Very Clear & Comprehensive**

Based on the feedback received, we made the following changes to the framework:

a) replaced the term "Mental Models of Integration" with "Integration Mindset" and modified the definition to include the types of mental models (Strategy and Roles);

b) removed "Beliefs" as a separate type of mental model and added a "belief/ perceptions" component to the two remaining mental model types (see Table 1);

c) edited several terms and definitions in the framework; and

d) developed additional content to include in the framework, primarily in the beliefs/perceptions components.

We would like your feedback on the revised terms, definitions and concepts in the framework. We will begin with the first aspect of an Integration Mindset: "Strategy Mental Model". Please review the information provided before answering the questions.

Mental Model Type	Definition
Strategy Mental Model	a conceptualization of what is being integrated and how, why and for whom it is being integrated

Strategy Mental Model: Knowledge Contents

Contents	Definition
Targets	functions, services, and/or organizations identified for integration
Clients	characteristics of the populations, patients and/or caregivers who will benefit from integration, and the nature of that benefit
Goals	primary objectives of integration, which may be related to costs, efficiency, quality of care, and/or patient outcomes
Long-term vision	how the services, programs or functions, and/or organizations will "look" or operate when fully integrated
Methods	approaches and enablers for achieving integration – which may be clinical, technological, patient or caregiver-centered, administrative, financial, organizational, governance and/or policy-related – and timeline for implementation
Evaluation	key performance dimensions and indicators for assessment of the integration activity

Strategy Mental Model: Beliefs/Perceptions Contents

Contents	Description
Consequences of integrating	expected outcomes (positive and negative) of the integration process
Appropriateness of selected strategy	extent of agreement with the selected targets, clients, goals, long-term vision, methods and evaluation approach for an integration activity
Integrity of decision-making processes	equitability and transparency of how decisions regarding integration are made
Aptitude for change	ability and willingness to implement the desired integration process

3. How clear are the definition and description of "Strategy Mental Model"?

☐	☐	☐	☐	☐	☐	☐
1	2	3	4	5	6	7
**Not Clear**			**Somewhat Clear**			**Very Clear**

4. A "Strategy Mental Model" consists of six types of knowledge and four types of beliefs. How comprehensive, or complete, are the contents of "Strategy Mental Model" (i.e. are there other strategy-related areas where similar ways of thinking are needed)?

☐	☐	☐	☐	☐	☐	☐
1	2	3	4	5	6	7
**Not Comprehensive**			**Somewhat Comprehensive**			**Very Comprehensive**

5. How useful is the concept of "Strategy Mental Model" to you in considering integration efforts and experiences?

☐	☐	☐	☐	☐	☐	☐
1	2	3	4	5	6	7
**Not Useful**			**Somewhat Useful**			**Very Useful**

6. Comments or Suggestions:

Below we describe the second aspect of an Integration Mindset: “Roles Mental Model”. Please review the information provided before answering the questions.

Mental Model Type	Definition
Roles Mental Model	a conceptualization of the individuals, groups, and organizations involved in integration, and their relationships

Roles Mental Model: Knowledge Contents[1]

Term	Definition
Competencies	the knowledge and skill sets (a) of each participating professional and (b) contained within each participating unit
Contributions	how participating professionals, units and patients/caregivers each contribute to patient health and well-being
Accountabilities	the activities and results that each participating professional and unit is responsible for
Interdependencies	how and to what extent the work of each participating professional, unit, and patient/caregiver depends on or is influenced by another
Communication	sources of information and how information flows at professional and unit hand-offs, including frequency and methods for contact; from the provider and patient/caregiver perspectives
Role impact	how integration efforts will influence or modify (or not) existing professional, unit, and patient/caregiver roles

[1] A fundamental requirement underlying these six areas of knowledge is a shared general awareness of “who is in and out” of the integration activity. Please note that depending on the nature and level of the integration activity (a) some of the contents and perspectives represented above may be less relevant and (b) “unit” can refer to programs, departments, organizations or sectors.

Roles Mental Model: Beliefs/Perceptions Content

Term	Description
Appropriateness of role structure	extent of agreement with the content and distribution of roles, including relative accountabilities and communication methods
Viability of hybrid identity	ability of individuals to simultaneously identify with their profession or occupation, their organization or sector, and the integration initiative (i.e. the team, partnership, network, etc.)
Recognition of shared responsibility	willingness to go beyond what one is obliged to do to support or contribute to the integration process
Importance of client involvement	extent to which the involvement of patients and their caregivers is considered necessary and beneficial to integration efforts

7. How <u>clear</u> are the definition and description of "Roles Mental Model"?

☐ 1	☐ 2	☐ 3	☐ 4	☐ 5	☐ 6	☐ 7
**Not Clear**			**Somewhat Clear**			**Very Clear**

8. A "Roles Mental Model" consists of six types of knowledge and four types of beliefs. How <u>comprehensive</u>, or complete, are the contents of "Roles Mental Model" (i.e. are there other roles-related areas where similar ways of thinking are needed)?

☐ 1	☐ 2	☐ 3	☐ 4	☐ 5	☐ 6	☐ 7
**Not Comprehensive**			**Somewhat Comprehensive**			**Very Comprehensive**

9. How <u>useful</u> is the concept of "Roles Mental Model" to <u>you</u> in considering integration efforts and experiences?

☐ 1	☐ 2	☐ 3	☐ 4	☐ 5	☐ 6	☐ 7
**Not Useful**			**Somewhat Useful**			**Very Useful**

10. Comments or Suggestions:

Below we define the overarching concept of an "Integration Mindset." Please review the definition before answering the questions.

Term	Definition
**Integration Mindset**	an individual's way of thinking about integration that is based on knowledge and beliefs regarding the **strategy** for achieving integration and the **roles** of those involved in the integration process

11. How clear is this term and definition to you?

☐	☐	☐	☐	☐	☐	☐
1	2	3	4	5	6	7
**Not Clear**			**Somewhat Clear**			**Very Clear**

12. An "Integration Mindset" consists of knowledge and beliefs in two key areas: Strategy and Roles. How comprehensive, or complete, is this definition (i.e. are there other areas where similar ways of thinking are needed)?

☐	☐	☐	☐	☐	☐	☐
1	2	3	4	5	6	7
**Not Comprehensive**			**Somewhat Comprehensive**			**Very Comprehensive**

13. Comments or Suggestions:

SECTION II: Are the Concepts Important?

Summary of Feedback from Round One

Average ratings for the importance of the key concepts in the framework:

0%	0.3%	0.7%	5%	15%	29%	50%
1	2	3	4	5	6	7
**Not Important**			**Somewhat Important**			**Very Important**

Below we ask you to rate the importance of the new concepts added to the framework.

Based on your experiences, how important do you think it is for those involved in an integration activity to have shared knowledge (i.e. information and awareness) in each of the following areas?

	**Not Important**			**Somewhat Important**			**Very Important**
**14. Evaluation** (key performance dimensions and indicators for assessment of the integration activity)	☐ 1	☐ 2	☐ 3	☐ 4	☐ 5	☐ 6	☐ 7
**15. Accountabilities** (the activities and results that each participating professional and unit is responsible for)	☐ 1	☐ 2	☐ 3	☐ 4	☐ 5	☐ 6	☐ 7
**16. Role impact** (how integration efforts will influence or modify (or not) existing professional, unit, and patient/caregiver roles)	☐ 1	☐ 2	☐ 3	☐ 4	☐ 5	☐ 6	☐ 7

Based on your experiences, how important do you think it is for those involved in an integration activity to have shared beliefs/perceptions in each of the following areas?

	Not Important			Somewhat Important			Very Important
**17. Consequences of integrating** (expected outcomes (positive and negative) of the integration process)	☐ 1	☐ 2	☐ 3	☐ 4	☐ 5	☐ 6	☐ 7
**18. Appropriateness of selected strategy** (extent of agreement with the selected targets, clients, goals, long-term vision, methods & evaluation approach for an integration activity)	☐ 1	☐ 2	☐ 3	☐ 4	☐ 5	☐ 6	☐ 7
**19. Integrity of decision-making processes** (equitability & transparency of how decisions regarding integration are made)	☐ 1	☐ 2	☐ 3	☐ 4	☐ 5	☐ 6	☐ 7
**20. Aptitude for change** (ability and willingness to implement the desired integration process)	☐ 1	☐ 2	☐ 3	☐ 4	☐ 5	☐ 6	☐ 7
**21. Appropriateness of role structure** (extent of agreement with the content & distribution of roles, including relative accountabilities & communication methods)	☐ 1	☐ 2	☐ 3	☐ 4	☐ 5	☐ 6	☐ 7
**22. Viability of hybrid identity** (ability of individuals to simultaneously identify with their profession, their organization or sector, & the integration initiative)	☐ 1	☐ 2	☐ 3	☐ 4	☐ 5	☐ 6	☐ 7

**23. Recognition of shared responsibility** (willingness to go beyond what one is obliged to do to support or contribute to the integration process)	☐ 1	☐ 2	☐ 3	☐ 4	☐ 5	☐ 6	☐ 7
**24. Importance of client involvement** (extent to which the involvement of patients & their caregivers is considered necessary & beneficial)	☐ 1	☐ 2	☐ 3	☐ 4	☐ 5	☐ 6	☐ 7

25. Comments or Suggestions:

SECTION III: How Useful is the Framework?

Summary of Feedback from Round One

On average,

- 40% rated the framework as *very useful* for interpretation, implementation and management; and
- 50% rated the framework as *very useful* for planning and evaluation.*

Feedback indicated the need to provide examples of how to apply the framework.

*Defined as a score of 6 or 7.

Response to Feedback

The framework is intended for measurement and discussion purposes. The aim is to identify which shared knowledge and beliefs facilitate integration and to enhance understanding of the multiple conceptualizations of integration. Below we offer examples of different applications of the framework; some examples require the development of a measurement tool for capturing and comparing integration mindsets.

Interpretation

Current or past integration efforts may be re-interpreted using the framework. For example, despite careful redesign of structures and processes, and a favourable environment, some integration efforts still collapse. Differences in integration mindsets may help partly explain such cases.

Planning

The framework can be used to direct and focus early discussions and planning efforts. For example, consideration for role competencies, contributions, and interdependencies may help inform integration design, particularly in terms of who needs to be at the table.

Implementation

The framework draws our attention to important knowledge content, some of which may be co-created or clarified, recorded, and formally agreed to during the implementation stage.

Management

Awareness of the extent to which integration mindsets are shared and where similarities and differences lie can help guide interventions by managers and leaders. For example, a lack of shared knowledge can be addressed through training and education, whereas a lack of shared beliefs will require more extensive and potentially long-term dialogue and negotiation in addition to changes to structures and incentives.

Evaluation

The extent to which integration mindsets are shared may help us assess the success and sustainability of an integration activity. As relationships develop and new work practices become embedded over time, knowledge and beliefs/perceptions become more congruent. So, we could use “shared integration mindsets” as one indicator, among many, of a successful and sustainable integration activity.

Based on information provided above and throughout this questionnaire,

Please rate the extent to which you think that the "Integration Mindsets" framework is <u>useful</u> for each of the following applications.

	Not Useful			Somewhat Useful			Very Useful
**26. <u>Interpreting</u> integration experiences**	☐ 1	☐ 2	☐ 3	☐ 4	☐ 5	☐ 6	☐ 7
**27. <u>Planning</u> integration activities**	☐ 1	☐ 2	☐ 3	☐ 4	☐ 5	☐ 6	☐ 7
**28. <u>Implementing</u> integration activities**	☐ 1	☐ 2	☐ 3	☐ 4	☐ 5	☐ 6	☐ 7
**29. <u>Managing</u> integration activities**	☐ 1	☐ 2	☐ 3	☐ 4	☐ 5	☐ 6	☐ 7
**30. <u>Evaluating</u> integration activities**	☐ 1	☐ 2	☐ 3	☐ 4	☐ 5	☐ 6	☐ 7

31. Comments or Suggestions:

32. Several modifications and additions have been made to the framework using participant feedback. Please rate the extent to which you think that the framework has been <u>improved</u>.

☐ 1	☐ 2	☐ 3	☐ 4	☐ 5	☐ 6	☐ 7
**No Improvement**			**Some Improvement**			**Big Improvement**

33. Comments or Suggestions:

<u>Thank you</u> very much for taking the time to complete this questionnaire.

We look forward to sharing the results with you; a detailed report will be sent to all participants during Summer 2013.

APPENDIX

Table of Essential Terms

Term	Definition
Belief	an opinion or internal feeling that something is true
Culture	the beliefs and behaviour patterns dominant among the members of an organization or profession
Integration	the process of bringing organizations and professionals together, with the aim of improving outcomes for patients through the delivery of integrated health and social care
Knowledge	information, awareness and understanding of someone or something
Mental Model	an individual’s way of thinking about how something works
Role	the rights, obligations, and expected behaviors of an actor in a particular social setting
Shared Mental Model	a way of thinking about how something works that is jointly held by multiple individuals (i.e. a common, but not necessarily identical, understanding)
Strategy	a plan of action designed to achieve a specific goal
