# Supplementary material for: A cognitive perspective on health systems integration: results of a Canadian Delphi study
Source: BMC Health Serv Res. 2014 May 19;14:222. doi: 10.1186/1472-6963-14-222 (PMC4066828; doi:10.1186/1472-6963-14-222)
Supplement: Additional file 3 — Summary of Sub-Group Differences by Respondents’ Level of Integration Experience, Current Workplace and Current Role. [file 1472-6963-14-222-S3.pdf]

Summary of Key Sub-Group Differences by Respondents' Level of Integration Experience

		**Micro-Level** (Mi, n=21)		**Meso-Level** (Me, n=43)		**Macro-Level** (Ma, n=26)	
	**Survey Item**[1]	Mean	SD	Mean	SD	Mean	SD
1st Round – all respondents	Usefulness of concept of "Roles Mental Model"[2]*	6.14>Me,Ma	0.96	5.37	1.46	5.08	1.55
	Usefulness of framework for managing integration*	5.52>Ma	1.63	5.07	1.18	4.50	1.48
		**Micro-Level** (Mi, n=16)		**Meso-Level** (Me, n=32)		**Macro-Level** (Ma, n=20)	
	**Survey Item**[1]	Mean	SD	Mean	SD	Mean	SD
1st Round – second round respondents	Usefulness of concept of "Roles Mental Model"[2]*	6.31>Me,Ma	0.87	5.31	1.50	5.15	1.50
	Usefulness of framework for managing integration*	5.81>Ma	1.33	5.22	1.29	4.66	1.42
	Comprehensiveness of definition of Strategy Mental Model**	5.75>Ma	0.86	5.44>Ma	1.01	4.85	0.81
	Usefulness of concept of "Strategy Mental Model"*	5.75>Ma	0.93	5.19	1.40	4.8	1.36
		**Micro-Level** (Mi, n=16)		**Meso-Level** (Me, n=32)		**Macro-Level** (Ma, n=20)	
	**Survey Item**[1]	Mean	SD	Mean	SD	Mean	SD
2nd Round – all respondents	Usefulness of the framework for managing integration*	6.13>Me	0.72	5.60	0.84	6.10>Me	0.97

[1] Only survey items for which there are significant differences across sub-groups are listed
[2] Later renamed "Relationships Mental Model"
A greater than (>) sign is used to denote significant group differences
*p<0.05
**p<0.01

Summary of Key Sub-Group Differences by Respondents' Current Role

		**Manager/Administrator or Consultant** (M, n=53)		**Clinician/Care Provider or Patient Advocate** (C, n=14)		**Researcher/Academic or Educator** (R, n=11)		**Policymaker** (P, n=12)	
	**Survey Item**[1]	Mean	SD	Mean	SD	Mean	SD	Mean	SD
1st Round – all respondents	Usefulness of framework for interpreting integration*	5.34>R	1.34	5.21	0.97	4.36	1.36	4.83	1.03
	Usefulness of framework for planning integration**	5.74>C,R	1.29	5.14	0.86	4.55	1.63	5.17	1.27
	Usefulness of framework for managing integration*	5.19>P	1.44	5.00	1.41	4.81	1.60	4.42	1.08
	Usefulness of framework for evaluating integration*	5.57>R	1.40	5.21	1.05	4.55	1.44	5.25	0.97
		**Manager/Administrator or Consultant** (M, n=41)		**Clinician/Care Provider or Patient Advocate** (C, n=11)		**Researcher/Academic or Educator** (R, n=8)		**Policymaker** (P, n=8)	
	**Survey Item**[1]	Mean	SD	Mean	SD	Mean	SD	Mean	SD
1st Round – second round respondents	Usefulness of framework for planning integration*	5.93>C,R	1.21	5.09	0.94	4.63	1.85	5.50	1.20
	Usefulness of framework for implementing integration*	5.51>C	1.53	4.64	0.92	5.25	1.39	5.00	1.20
2nd Round – all respondents	N/A								

[1] Only survey items for which there are significant differences across sub-groups are listed.
A greater than (>) sign is used to denote significant group differences
*p<0.05
**p<0.01

Summary of Key Sub-Group Differences by Respondents' Current Workplace[1, 2]

		**Coordinating or Advisory Body** (CA, n=14)		**Hospital** (H, n=10)		**Community, Home, Long-Term, Care** (C, n=9)		**Ministry / Government** (M, n=7)		**RHA/DHA or LHIN** (R, n=8)		**Primary Care** (P, n=7)		**Professional Association or College** (Pr, n=5)	
	**Survey Item**[3]	Mean	SD	Mean	SD	Mean	SD	Mean	SD	Mean	SD	Mean	SD	Mean	SD
1st Round – all respondents	N/A														
1st Round – second round respondents	N/A														
2nd Round – all respondents	Usefulness of concept of "Roles Mental Model"*	4.64	1.01	5.90 >CA	0.88	5.33 >CA	1.00	5.29	1.80	6.25 >CA	0.71	6.00 >CA	0.82	6.20 >CA	0.84
	Usefulness of framework for interpreting integration*	5.00	0.96	5.80	0.92	6.11 >M	0.60	4.86	1.07	5.88 >CA >M	0.64	5.89	0.69	6.40 >CA >M	0.55

[1] "University/Research Institute" is not included in the table because responses from this sub-group were not significantly different from those of any other sub-group

[2] Only sample size for the second round is reported in the table

[3] Only survey items for which there are significant differences across sub-groups are listed

[4] Later renamed "Relationships Mental Model"

A greater than (>) sign is used to denote significant group differences

*$p<0.05$
